# Cell membrane rupture: a novel test reveals significant variations among different brands of tissue culture flasks

**DOI:** 10.1186/s13104-021-05453-7

**Published:** 2021-01-26

**Authors:** Ruy Tchao

**Affiliations:** Department of Pharmaceutical Sciences, University of the Sciences, Philadelphia, PA 19104 USA

**Keywords:** Polystyrene, Tissue culture, Cell membrane stability

## Abstract

**Objectives:**

Loss of cytoplasmic molecules including protein controls, due to cell membrane rupture can cause errors and irreproducibility in research data. Previous results have shown that during the washing of a monolayer of cells with a balanced salt solution, the fluid force causes cell membrane rupture on some areas of the flasks/dishes. This fact shows the non-uniformity of the polystyrene surface in terms of cell culture. There is at present no simple test to monitor that surface. This paper presents a novel biologically based assay to determine the degree of heterogeneity of flasks supplied by various manufacturers.

**Results:**

This paper shows that significant variation exists in polystyrene surface heterogeneity among several brands of tissue culture flasks, varying from 4 to 20% of the flask surface. There is also large variability within the production lot of a manufacturer. The assay method involves loading the cells with a cytoplasmic fluorescent marker that is released upon cell membrane rupture. Cell membrane rupture also causes the loss of marker proteins such as GAPDH used in Westernblots. This novel assay method can be used to monitor the batch consistency and the manufacturing process of flasks/dishes. It may also be used to test new biomaterials.

## Introduction

The physical microenvironment in vitro such as the substratum can affect cell adhesion [1, 2], cell motility [3] and fluid force can influence endothelial cell signal transduction [4].

Polystyrene (PS) is the material used ubiquitously in cell culture [5]. We have reported that application of a fluid force, such as the rinsing of cells after the removal of medium, on certain areas of the flask or dishes, the plasma membrane of cells will rupture immediately while the cells are still adherent to the PS surface [6]. This effect appears to be related to the stress in the PS polymer that developed during the manufacturing process and can be demonstrated with birefringence pattern in the plastic [7]. This fluid shear effect was initially observed with the squamous cells line, NBT-II and several epithelial cell lines. Some epithelial cells lines do not respond to fluid shear. For example, MDCK cells that have tight junctions do not respond. MCF10A responds to fluid shear similarly to NBT-II cells but MDA MB231 cell line does not respond to fluid shear (see Additional file 1).

For molecular and biochemical analysis of the cultured cells, after the removal of the culture medium, cells are generally rinsed with a buffered salt solution such as PBS or HBSS. Therefore, a concern is that the rinsing process can cause the loss of house-keeping marker proteins such as GAPDH used in Westernblots. Specific target molecules in the cytoplasm may also be lost, leading to variability in repeating experimental results, as well as the irreproducibility of the results from another lab that uses different flasks/dishes.

Variability in PS surface has been demonstrated in PS flasks and dishes by physical measurements [8]. This paper describes a novel biologically based method to determine quantitatively the percent of the heterogeneity of the PS surface in tissue culture flasks and dishes that contribute to cell membrane rupture due to fluid shear force. This novel method can be helpful for the end user to compare the consistency of batches of plastic ware. This method can also be used by PS manufacturers to monitor the quality of their production, including any changes in the manufacturing process and for testing new biomaterials.

## Main text

### Methods and materials: details are presented in Additional file 2

Samples of T-25 flasks were obtained from selected manufacturers (Chemglass, Corning, Falcon, Greiner, Nunc, Santacruz, Sarstedt, TPP).

*Protocol of fluid shear in flasks: Please see Additional file* *3**for details. It is beneficial for readers to access the additional files to completely appreciate this paper.* Briefly, cells grown in T25 flasks from various manufacturers were incubated with 2 μM CalceinAM for 60 min. After a first rinse with *cold HBSS*, then a subsequent *rinse with warm HBSS to cause the fluid shear effect*, the supernatants are collected for fluorescence measurement. The flask is then incubated with TritonX100 to release the remaining fluorescence in cells. The percentage of fluorescence released by fluid shear to the total fluorescence is calculated and expressed as the percent of heterogeneity of the flask surface.

### Results

**Fig. 1:**
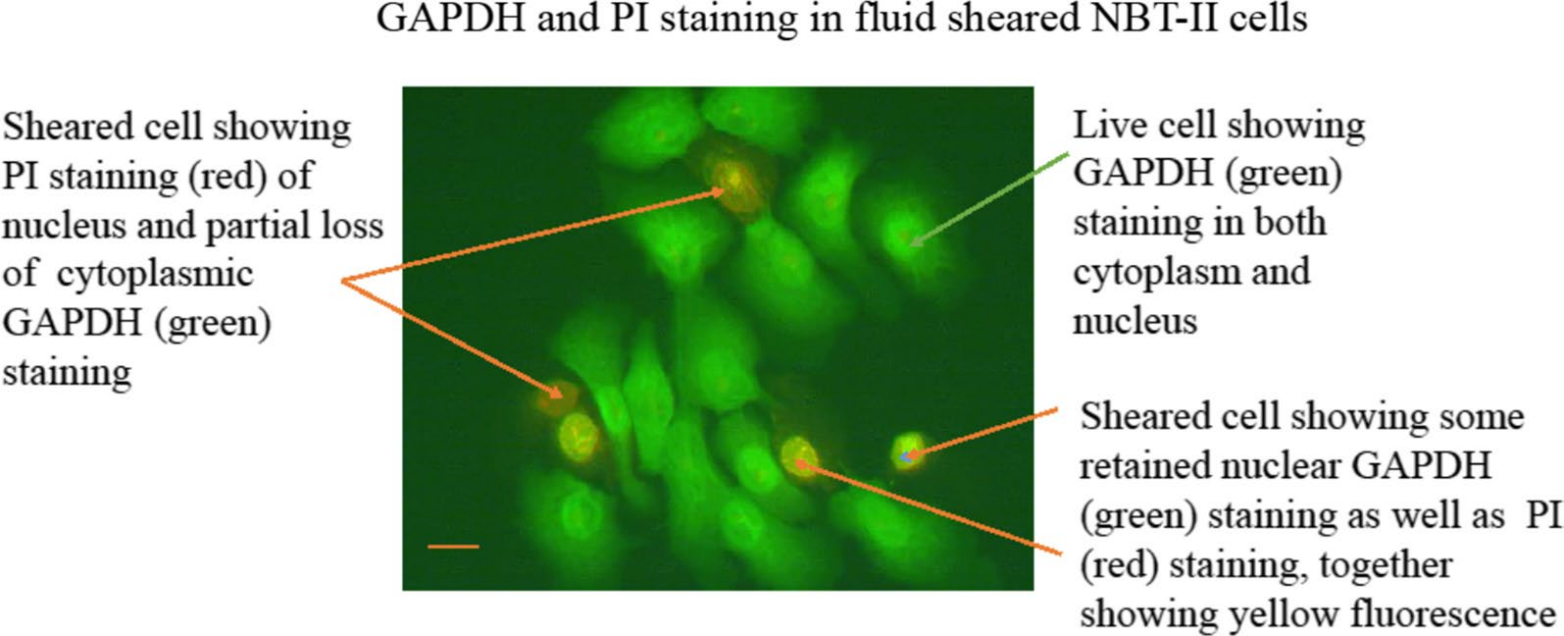
GAPDH and PI staining in Fluid Sheard NBT-II cells. After fluid shear, the cells were stained with propidium iodide, then fixed and stained with Alexa488 conjugated anti GAPDH antibody. Bar at left bottom = 10 μm

Initially experiments using Alexa488 labelled antiGAPDH showed that after fluid shear, there is some loss of the marker protein from the cytoplasm in some cells, but in other cells, GAPDH may be retained to varying degree in the nucleus of the cells as shown in Fig. 1.

Since not all cells lose the marker proteins GAPDH equally throughout the culture, and GAPDH may be retained in the nucleus, GAPDH cannot be used to quantify the effect of fluid shear. *Therefore, a smaller molecule that is not bound inside the cells will be a better choice to quantify the fluid shear effect.*

CalceinAM, a non-fluorescent molecule is easily taken up by live cells, and inside the cell, *CalceinAM is converted by non*-*specific esterases into the fluorescent molecule Calcein* [9].

**Fig. 2:**
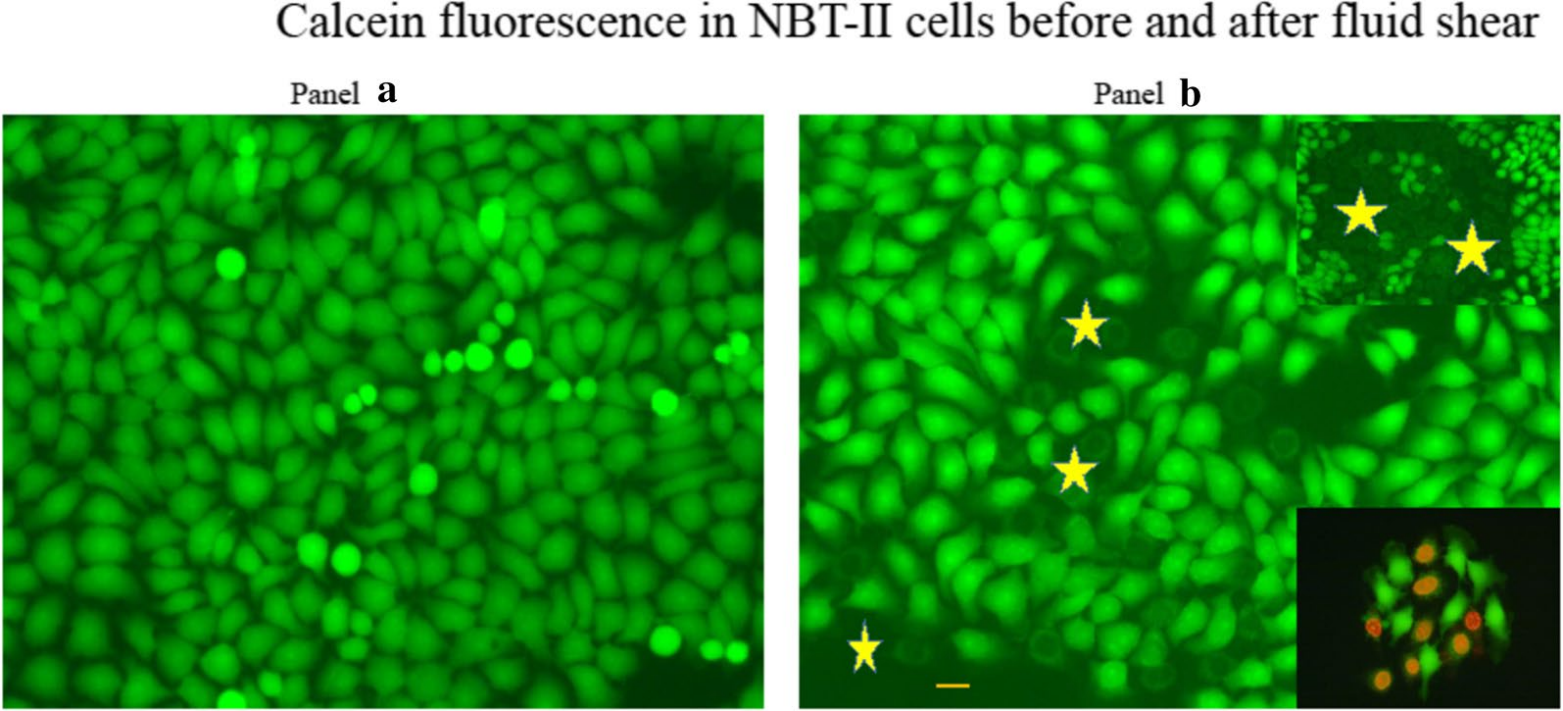
Calcein Fluorescence in NBT-II cells Before and After Fluid Shear. Panel **a** shows cells preloaded with CalceinAM, giving green fluorescence of Calcein in the cytoplasm. Panel **b** shows fluid shear cause some cell membrane to rupture with loss of the green cytoplasmic fluorescence (marked by star). The membrane ruptured cells appear as “ghost” cells inter-dispersed among live cells. Occasionally there are large patches of ruptured cells as shown in the upper inset in Panel **b**. Ruptured cells can be stained with propidium iodide as shown in the lower inset in Panel **b**. Bar = 10 μm

Preliminary experiments show qualitatively that CalceinAM loaded cells show green fluorescence, and after fluid shear, the ruptured cells lose the Calcein green fluorescence, and the nucleus can be stained with propidium iodide as shown in Fig. 2. The staining of live cells with Calcein and dead cells with propidium iodide is the basis of the Live/Dead Assay^®^ developed by Invitrogen.

As illustrated in Fig. 2 panel B inset, the close juxtaposition of live and ruptured cells suggests that the heterogeneity of polystyrene is at the micro or nano level. This presents a challenge to the detection of polystyrene surface irregularity by physical methods.

Calcein uptake by NBT-II cells is dependent on cell number and concentrations of CalceinAM. There is a linear relationship of uptake between time and normalized cell number. *Please see Figure* *3 in Additional file* 4*for the kinetics of CalceinAM uptake.*

Triton X100, 0.1% is used to extract total fluorescence in cells. The data showing 100% efficacy of Triton extraction are presented in Additional file 4.

A comparison of T-25 flasks from various manufacturers is shown in Table 1.

**Table 1:** Fluid shear effect of NBT-II cells grown in T-25 of various manufacturers

Manufacturer	Cat #	Lot #	% Fluid shear effect ± sem
Chemglass(CellTreat)	229331	110723-218	10.5 ± 0.5*
Corning (regular)	3056	09815045	14.1 ± 0.6*
Corning (Cell-Bind)	3289	22005005	8.7 ± 0.5
Falcon	353108	5249004	13.6 ± 1.4*
Greiner	690170	08050140	11.3 ± 0.2*
Nunc	136196	136985	20.6 ± 0.3*
SantaCruz	SC-200262	110423-218	11.8 ± 0.5*
Sarstedt	831810	6065081	13.4 ± 1.1*
TPP	90025	20150141	4.5 ± 1.3

The results shown in Table represent 3 separate runs on each type of T-25 flasks. Therefore, the sem represent n = 3. For each run, 3–4 flasks of each brand are used

* Indicates that fluid shear effect in flask is significantly greater (p < 0.05) than the non-shear value

The results show that there is considerable difference, varying from 4.5 to 20.7% of the total T-25 flask surface among the manufacturers. *Please see Additional file* 3*for details of fluid shear protocol and associated data*. A specialized treatment to alter the PS surface charges (CellBind^®^) did not prevent the fluid shear effect. In comparison to NBT-II cells, MDCK cells do not exhibit fluid shear effect as shown in Table 2.

**Table 2:** Fluid shear effect of MDCK cells grown in T-25 flasks

Manufacturer	% shear ± sd
Corning	2.4 ± 0.4
Greiner	3.3 ± 0.4

5 Flasks of each manufacturer are used. The fluids shear effect is not statistically significant

### Discussion

It is a long and generally held view by researchers that for tissue culture studies, one should not change the manufacturer of tissue culture flasks and dishes lest it might affect the reproducibility of the results. During manufacturing of tissue culture flasks, some factor or factors associated with the injection mold process can cause polystyrene surface heterogeneity, resulting in different PS surfaces produced among the manufacturers.

To date, there is not a simple method to determine the heterogeneity of PS surface. This paper describes a novel biological assay based on cell membrane rupture due to fluid shear. Because cell membrane rupture appears after the application of a fluid shear force equivalent to rinsing the cell culture with a balanced salt solution, the membrane rupture cannot be due to an osmotic effect. Ruptured cells remain adherent but cytoplasmic content, including cytoplasmic molecules of interest such as signal transduction proteins, could be released. Quantitative measurement of protein loss such as GAPDH would be desirable. However, the loss of GAPDH would have to be linked quantitatively to cell death/membrane rupture. Calcein in cells is not bound, and dead cells (membrane ruptured cells) do not retain Calcein [9]. Therefore, the fluorescence measurement of Calcein loss by cells in fluid shear will be more direct, quantitative and simpler. This paper shows that the amount of the fluid shear rupture of cell membrane is variable among tissue culture flasks of different manufacturers causing variable results when different manufacturers for PS flasks and dishes are used. In addition, cells such as MDA-MB231 do not respond to fluid shear, whereas MCF-10A cells respond to fluid shear. As one compares the expression of molecules in these two cell lines, errors may occur.

Freedman et al. [10] showed that biological reagents and reference material accounted for 36.1% of the total cost of irreproducibility in pre-clinical research. Tissue culture technique is used widely in preclinical research. Battiston et al. [11] have shown that tissue culture polystyrene from different manufacturers differ considerably in protein binding. However, standardization in the PS used in flasks and dishes does not exist. This assay using cell membrane rupture and the release of Calcein in an epithelial cell line can be used to monitor the manufacturing of tissue culture flasks as well as the suitability of biomaterial for cells [12].

The mechanisms of cell responses to the physical and chemicals nature of the substratum are complex [13, 14]. The exact mechanism involved in cell membrane rupture upon fluid shear on certain areas of the PS surface is not known. PS surface charge may not be the only factor as shown in Table 1 with Corning CellBind flasks [15].

Figure 2 Panel B illustrated the close juxtaposition of live and dead cells, suggesting that the sensing of PS surface by cells may be at micro-meter or nano-meter level. Previous studies [7] have shown that in the edges of the flasks and dishes, and in areas related to the injection port, there is strong birefringence under cross-polarized light, suggesting that PS molecular orientation may contribute to the sensitivity of cells to fluid shear. We have also shown that stress force applied to a polystyrene disk increases fluid shear effect on the disk [7]. As the PS polymer solidifies, shrinkage can affect the dimension of the final product and also has a large effect on the residual stress distribution of the product [16, 17]. The molecular orientation of the PS polymer surface can be modified and changed by rubbing [18, 19].

The integrity of cell membrane under fluid shear may have relevance in vivo such as the expression of anti-or pro-inflammatory molecules by the endothelium under various vascular flow patterns leading to propensity for atherogenesis [20]. In cancer metastasis, tumor cells have been shown not to survive the hemodynamic shear force [21]. Therefore, adherence to the endothelium may alter the tumor cell response to fluid shear force.

### Conclusion and significance

This paper describes a novel biologically based test for tissue culture flasks and dishes. As the rinsing of a cell culture with a balanced salt solution is routinely done before biochemical analysis, the rupture of some cells that release cytoplasmic content could lead to possible errors in research data. Products from various manufacturers differ significantly in their surface heterogeneity. This can result in irreproducibility of data among research laboratories.

## Limitations

The mechanism of this fluid shear effect on cells is not known. Because the appearance of birefringence of the flasks and the response of NBT-II cells to stressed polystyrene, we speculated that this fluid shear effect of cell rupture is due to the molecular orientation of the polystyrene polymer. Detailed analysis of the PS surface using atomic force microscopy or IR spectroscopy are needed to identify the mechanisms. However, it is difficult to relate the result of a physical measurement to the specific cell that shows the response, whereas an adjacent cell may not show membrane rupture. In order to quantify the release of cytoplasmic marker proteins from the sheared cells, ELISA assay for the specific proteins such as GAPDH would need to be performed.

## Supplementary information

**Additional file 1.** Absence of fluid shear effect on MDA-MB231 cells.

**Additional file 2.** Methods and Materials.

**Additional file 3.** Fluid shear procedure.

**Additional file 4.** Kinetics of CalceinAM uptake.

## Data Availability

The raw data required to reproduce these findings are available to download from:https://drive.google.com/drive/u/0/folders/13uj_LdCt4JQD0HTs06S6P2959Dw_pOn5.

## Abbreviations

DMSO: Dimethylsuphoxide
HBSS: Hanks balanced salt solution
GAPDH: Glyceraldehyde 3-phosphate dehydrogenase
MDCK: Madin Darby Canine kidney
NBT-II: Nara bladder tumor II
PBS: Phosphate buffered saline
PI: Propidium iodide
PS: Polystyrene
