# Supplementary material for: Cell membrane rupture: a novel test reveals significant variations among different brands of tissue culture flasks
Source: BMC Res Notes. 2021 Jan 26;14:38. doi: 10.1186/s13104-021-05453-7 (PMC7836507; doi:10.1186/s13104-021-05453-7)
Supplement: Supplementary file 1 — Additional file 1. Absence of fluid shear effect on MDA-MB231 cells. [file 13104_2021_5453_MOESM1_ESM.docx]

**Additional File #1 (Tchao)**

**Fluid shear effect on MCF10A and MDA MB231 cells**

These images present a cautionary note that comparing two different cell lines for expression of small molecules in the cytoplasm may yield uncertain results because if one cell line such as ***MCF 10A that responds to fluid shear*** with cell membrane rupture while another cell line such as ***MDA MB231 that does not respond to fluid shear****.*
