# Supplementary material for: Cell membrane rupture: a novel test reveals significant variations among different brands of tissue culture flasks
Source: BMC Res Notes. 2021 Jan 26;14:38. doi: 10.1186/s13104-021-05453-7 (PMC7836507; doi:10.1186/s13104-021-05453-7)
Supplement: Supplementary file 2 — Additional file 2. Methods and Materials. [file 13104_2021_5453_MOESM2_ESM.docx]

**Additional file # 2 (Tchao)**

**Methods and Materials:**

NBT-II cells are routine culture in DMEM/F12 medium supplemented with 10% fetal bovine serum, all obtained from ThermoFisher.

HanksBalanced salt solution (HBSS) **with Ca++ and Mg++ without phenol red**, is obtained from ThermoFisher (cat number 14025092).

CalceinAM was obtained from Lifesciences as solids in 50µg/vial. Each vial is dissolved in 50µL DMSO to give a stock solution of 1mM concentration. For incubation with cells, the stock solution is diluted to 0.5-4 µM in HBSS. Preliminary experiments using 0.5µM CalceinAM have shown that DMSO from 0.1-0.4% do not affect CalceinAM uptake by cells.

CyQuant assay kit was purchased from Lifesciences , InVitrogen ® CyQUANT Cell Proliferation Assay Kit.

Anti GAPDH labelled with Alexa488 was purchased from ThermoScientific (cat #MA5-15738-D488) .

Zeiss Axioscope equipped with Polaroid color imaging system is used to record fluorescent images. NIH ImageJ program is used to merge fluorescent images.

Fluorescence is quantitatively measured in a plate reader, Cyto2300 at excitation 485nm and emission 590 nm.
