# Supplementary material for: Cell membrane rupture: a novel test reveals significant variations among different brands of tissue culture flasks
Source: BMC Res Notes. 2021 Jan 26;14:38. doi: 10.1186/s13104-021-05453-7 (PMC7836507; doi:10.1186/s13104-021-05453-7)
Supplement: Supplementary file 3 — Additional file 3. Fluid shear procedure. [file 13104_2021_5453_MOESM3_ESM.docx]

**Additional file #3 (Tchao)**

**Fluid shear Procedure applied to T-25 flasks:**

Based on the preliminary results on CalceinAM loading, the following procedure has been adopted to measure the percentage of shear in T-25 flasks by various manufacturers.

Culture medium is aspirated from the T-25 flasks. ***2ml cold HBSS*** is added to rinse the cells and then aspirated. Previous experiments have shown that medium (DMEM/F12 + 10FBS) aspiration does not cause fluid shear.  ***Cold HBSS does not produce the fluid shear effect of membrane rupture in cells*** *[6,7].*

2ml CalceinAM, 2µM, is added to each T-25 flask and incubated at 37^o^C for 60 minutes.

At the end of CalceinAM incubation, ***2 ml Cold HBSS*** is added to each flask and extracellular CalceinAM is removed by aspiration, and the flasks are ***rinsed twice with 2 ml cold HBSS*** each time. The supernatants were collected for fluorescence measurement. Each rinse solution has been found to be negligible.

The ***second rinse of 2ml HBSS*** is kept for fluorescence measurement, that serves as the background for the specific flask to calculate for the ***non-shear %*** of the flask.

Then*,* ***warm HBSS 2ml is added*** and the flask is ***incubated at 37^o^C*** for 10 minutes to equilibrate the warm HBSS solution and the flask. Fluid shear is applied to the cells by rocking the flask back and forth, tilting the flask by 90degree each time, the *entire* ***HBSS fluid will wash over the cells****.* The fluid is collected for fluorescence measurement, representing the ***fluorescence from sheared cells****.* After the removal of the “fluid shear” solution, 2 ml of 0.1% Triton X100 in HBSS neutralized to pH 7.2, is added and incubated at room temp for 10 minutes to release the total fluorescent Calcein in the remaining cells. ***The Triton solution is collected for fluorescence measurement.***

All fluorescence measurements were done in a fresh 24 well plate.

The following formula is used to calculate the percent of shear in each flask :

Sheared cell washings + Triton X100 released fluorescence = Total fluorescence

Fluid Sheared cell solution fluorescence / Total fluorescence = % heterogeneity on PS surface

For each run, 3-4 flasks of each brand are used. The experiments are repeated 3x (n=3) for statistical analysis of significance.
