# Supplementary material for: Cell membrane rupture: a novel test reveals significant variations among different brands of tissue culture flasks
Source: BMC Res Notes. 2021 Jan 26;14:38. doi: 10.1186/s13104-021-05453-7 (PMC7836507; doi:10.1186/s13104-021-05453-7)
Supplement: Supplementary file 4 — Additional file 4. Kinetics of CalceinAM uptake. [file 13104_2021_5453_MOESM4_ESM.docx]

**Additional file #4 (Tchao)**

**Data to characterize the kinetics of CalceinAM uptake by NBT-II cells**

**Triton extraction data :**

In order to measure the total Calcein fluorescence in each flask, the cells were extracted with 0.1% neutral TritonX100. To test the efficiency of extracting total Calcein fluorescence in cells, cells are grown in a 24-well plate to 70-80% confluent cultures. Cells are then loaded with CalceinAM (2-5 µM) in 1ml HBSS. The Calcein fluorescence is measured in a plate reader. After the removal of the CalceinAm incubation solution, the wells are rinsed with cold HBSS. The total Calcein fluorescence in the cells is extracted completely with Triton X100 as represented by percent of Calcein fluorescence measured in the 24-well plate and the results are shown in Table 2.

Table 2: Triton extraction of preloaded CalceinAM fluorescence.

|  | Triton extraction % | SD |
| --- | --- | --- |
| Expt 1 | 98.8 | 0.04 |
| Expt 2 | 102.7 | 0.09 |
| Expt 3 | 95.0 | 0.13 |

Table 2 legend: Triton extracted fluorescence was compared to the fluorescence in monolayer cells in 24 well plates, after 60 minutes incubation with 2µM CalceinAM. Each experiment consists of 12 wells of cells. After rinsing the cells with cold HBSS, 1ml 0.1% neutralized Triton x100 was added. After incubation at room temp for 10 minutes, the triton solutions were removed into fresh wells for fluorescence measurements.
